# Growth and stabilization of induced seismicity rates during long-term, low-pressure fluid injection

**DOI:** 10.1098/rsta.2023.0183

**Published:** 2024-08-09

**Authors:** James P. Verdon, Benjamin Pullen, Germán Rodríguez-Pradilla

**Affiliations:** ^1^ School of Earth Sciences, University of Bristol, Wills Memorial Building, Queen’s Road, Bristol BS8 1RJ, UK

**Keywords:** induced seismicity, statistical seismology, earthquake forecasting

## Abstract

We examine the temporal evolution of sequences of induced seismicity caused by long-term fluid injection using a compilation of over 20 case studies where moderate magnitude (*M* > 3.0) induced events have been recorded. We compare rates of seismicity with injection rates via the seismogenic index and seismic efficiency parameters, computing both cumulative and time-windowed values. We find that cumulative values tend to accelerate steeply as each seismicity sequence initiates—most cases reach a value that is within 0.5 units of their maximum value within 1–3 years. Time-windowed values tend to increase to maximum values within 25%–35% of the overall sequence, before decreasing as levels of seismicity stabilize. We interpret these observations with respect to the pore pressure changes that will be generated in highly porous, high permeability reservoirs. In such situations, the rate of pore pressure change is highest during the early phases of injection and decreases with time. If induced seismicity scales with the rate of deformation, which in turn is controlled by the rate of pore pressure change, then it is to be expected that induced seismicity is highest during the early phases of injection, and then decreases with time.

This article is part of the theme issue ‘Induced seismicity in coupled subsurface systems’.

## 1. Introduction

Induced seismicity has proved to be a major issue associated with industrial activities that involve subsurface fluid injection, such as wastewater disposal (WWD), hydraulic fracturing (HF), enhanced geothermal systems, natural gas hydrogen storage (NGS) and carbon capture and storage (CCS). The increasing scale and utilization of these industries have led to growing concern regarding induced seismicity hazards as more cases of fluid injection-induced seismicity have occurred. Larger induced seismic events, such as the *M* 5.6 Prague and *M* 5.8 Pawnee sequences in Oklahoma [1,2], the Pohang sequence in South Korea (*M* 5.5 [3]) and sequences in the Sichuan Basin, China (*M* 5.7 [4]), have proved capable of causing damage to nearby buildings and infrastructure. Smaller induced events, even if of insufficient magnitude to cause damage, nevertheless often provoke significant public concern [5].

As such, there is a need to better understand the physical processes that take place as subsurface injection impinges on tectonic faults, triggering induced seismicity. By doing so, we may be able to improve our estimations of induced seismicity hazard during the lifetime of injection operations. Improved estimates of hazard can in turn be used to develop appropriate regulations and mitigation strategies to control and mitigate induced seismicity.

### (a) Seismic efficiency and seismogenic index

The rate of earthquake occurrence, *λ*, is given by [6]:


(1.1) $$\lambda =\frac{r\overset{˙}{\tau }}{{\overset{˙}{\tau }}_{r}},$$

where $\overset{˙}{\tau }$ is the shear stressing rate and *r* is the earthquake rate at a reference stressing rate ${\overset{˙}{\tau }}_{r}$ . If we assume that during the operation of a given injection facility, the stressing rate caused by the injection is much larger than the background tectonic stressing rate (which can be taken as the reference condition for our purposes here), then the rate of induced seismicity will scale linearly with the stressing rate produced by the injection. In turn, we might expect the stressing rate to scale linearly with the injection rate (we examine this assumption further in our discussion). If the above assumptions are true, it is to be expected that the rate of induced seismicity occurrence will scale to the injection rate.

This expectation is manifest in two parameters that are commonly used to quantify the relationship between injection rates and the resulting induced seismicity: seismogenic index [7] and seismic efficiency [8].

The seismogenic index, *S*_I_ [7], relates the number of induced earthquakes, *N*_E_, larger than a magnitude *M*, to the injected volume Δ*V*:


(1.2) $$S_{I}=\log\left(\frac{N_{E}}{\Delta V}\right)+bM,$$

where *b* is the Gutenberg and Richter [9] *b* value. Typically, the minimum magnitude of completeness, *M*_MIN_, is used as the reference magnitude *M*.

The seismic efficiency, *S*_EFF_ [8], relates the cumulative release of seismic moment, Σ*M*_O_, to the injected volume:


(1.3) $$S_{EFF}=\;\frac{\Sigma M_{0}}{\mu \Delta V},$$

where *μ* is the shear modulus of the rock in which the seismicity is taking place. Again, typically the cumulative moment is summed only for events larger than *M*_MIN_. To facilitate comparisons between *S*_EFF_ and *S*_I_, since *S*_I_ is defined as the logarithm of seismicity rate versus volume (equation (1.2)), we also define a similar logarithm for the moment-based term *S*_EFF_:


(1.4) $$S_{E}=\log_{10}S_{EFF}.$$

Since the logarithm of the seismic moment scales with 1.5 × *M*_W_, the formulation for *S*_I_ (equation (1.2)) implicitly posits a scaling between seismic moment and injected volume of Σ*M*_O_∝ Δ*V* ^3/2^, whereas for *S*_EFF_ the scaling is linear, Σ*M*_O_∝ Δ*V* ^1^. There remains debate over what scaling between induced seismicity moment and injection volume might be more appropriate [10–12], and it can be difficult to constrain empirically because in practice the measured constant of proportionality between these terms may evolve during the course of injection [13].

We note that the formulations for *S*_I_ and *S*_EFF_ above do not impose any sort of volume-based cap on maximum magnitudes, as per McGarr [12]. The volume-based cap assumes that the strain released by the induced seismicity is solely or predominantly that imposed by the subsurface operations [12]; as such *S*_EFF_ cannot exceed a value of 1, since the total seismic moment release cannot exceed the total amount of deformation imparted by the injection. Some researchers make a distinction between ‘induced’ and ‘triggered’ seismicity where for induced seismicity the bulk of the strain released by the seismicity is imparted by the subsurface operations, whereas for triggered seismicity the subsurface operations serve to nucleate the seismicity but the bulk of the strain that is released is tectonic strain accumulated over geological timeframes [14].

However, various observations pertaining to injection-induced seismicity suggest that most cases should be regarded as ‘triggered’ under the above definition (though robust discrimination between the two types is often challenging, and in many cases, the reality may lie somewhere between the two endmembers). Injection-induced seismicity occurs on pre-existing tectonic faults [15], and focal mechanisms are usually consistent with the *in situ* tectonic stress regime [16], implying that tectonic strain is likely being released. Moreover, there are numerous examples where the maximum magnitudes have exceeded the limits imposed by the McGarr cap [3,17]. Therefore, we use equations (1.2)–(1.4) to posit a linear scaling between earthquake rates and injected volumes, based on the reasonable assumption that the stressing rate imposed by injection will scale linearly with injection volume. However, we do not impose any volume-based limits to this scaling as per McGarr [12], meaning that *S*_EFF_ values can exceed *S*_EFF_ > 1 where necessary.

### (b) Induced seismicity hazard forecasting

Both *S*_I_ and *S*_E_ can be used to forecast induced seismicity hazards. If it is assumed that the scaling between volume and induced seismicity rate stays constant then we can use these parameters to calculate the number of earthquakes or the cumulative seismic moment that will be generated by the injection of some future volume of fluid (e.g. the total planned injection volume for a well). From equation (1.2), the total number of earthquakes that will be generated by a total injection volume *V*_T_ is given by:


(1.5) $$N_{E}=V_{T}10^{S_{I}-bM},$$

from which the expected largest magnitude event, *M*_MAX_, can be computed, assuming the seismicity follows a Gutenberg–Richter (G–R hereafter) distribution:


(1.6) $$M_{MAX}=\frac{\left(S_{I}-\log\left[\frac{-\ln\chi }{V_{T}}\right]\right)}{b},$$

where *χ* is the probability that this magnitude is not exceeded.

From equations (1.3) and (1.4), the total seismic moment released is given by:


(1.7) $$\Sigma M_{0}=\;\mu V_{T}10^{S_{E}}.$$

The size of the expected largest event can then be estimated from the cumulative seismic moment release [12]:


(1.8) $$M_{MAX}=\frac{\frac{2}{3}b}{1-\frac{2}{3}b}\;\Sigma M_{O}.$$

This approach to induced seismicity forecasting has been used to make operational real-time forecasts at some sites, such as during enhanced geothermal stimulation at the Helsinki St1 Deep Heat project [18], at the Weyburn Carbon Capture and Storage Project [19], during HF in the Preston New Road shale gas wells in Lancashire, UK [13,20] and forecasting the impacts of injection rate changes on induced seismicity in Oklahoma [21].

### (c) Geomechanical implications of time-varying induced seismicity rates

The performance of these forecasting models hinges upon the assumption that *S*_E_ and/or *S*_I_ remain constant during fluid injection. Dinske and Shapiro [22] presented *S*_I_ data for a selection of case studies, primarily comprising short-term HF and geothermal stimulation operations, which showed relatively constant values during injection for each site (with values varying significantly, by as much as 10 orders of magnitude, between different sites). However, there are reasonable geomechanical arguments that could be invoked to explain why one might expect *S*_E_ and *S*_I_ to vary during injection at a given site:

— As a perturbation spreads laterally from an injection well, it may encounter faults that are more seismogenic (i.e. closer to their critical stress point), or a volume of rock that contains more faults. This will result in more reactivation and an increase in induced seismicity relative to a constant injection rate [20].
— It is widely accepted that larger magnitude-induced seismicity predominantly releases tectonic strain that has built up over geological time [23]. Given the relative timescales involved, there is no opportunity for tectonic stresses to be reloaded during injection. Therefore, if faults have a limited budget of tectonic strain, the rates of induced seismicity would reduce once a significant portion of that budget is depleted [24].
— As described in equation (1.1), the linear scaling between injection volumes and seismicity is an outcome of the assumption of a linear scaling between stressing rate and the rate of seismicity. While this would seem to be a reasonable assumption, there is no physical reason why this must be true in all scenarios, and changes in the scaling between stressing rate and seismicity would likely result in changes in the observed relationship between injection and seismicity.
— Moreover, in addition to a fixed scaling between stressing rate and seismicity, a further assumption is that there is a linear scaling between the injection volume and the resulting stressing rate. However, this assumption may not always be appropriate. For example, with injection into a laterally unbounded, high porosity/permeability formation the pore pressure will initially increase but will then evolve towards a steady-state condition. At this point, continued injection will produce perturbations that are smaller and smaller, and so the rate of induced seismicity might be expected to decrease.

### (d) Study objectives

Watkins *et al*. [25] examined sequences of WWD-induced seismicity (WWD-IS) and found that the largest events in each sequence tended to occur during roughly the first one-third of the overall seismicity sequence. This observation was in stark contrast to the observations made by Verdon and Bommer [26] for HF-induced seismicity, where the largest events were found to be systematically towards the ends of the observed sequences. Watkins *et al*. [25] did not compile any injection data, and so they were not able to rule out the possibility that the changes in the levels of seismicity they observed were driven solely by changes in injection rates.

The objective of this study is to examine how the scaling between seismicity and injection volume, as characterized by the *S*_I_ and *S*_E_ parameters, evolves during subsurface injection operations. Any systematic variability that we observe may prove to be informative with respect to the underlying geomechanical and tectonic processes that take place as induced seismicity is generated.

Furthermore, as described in equations (1.6) and (1.8), the *S*_I_ and *S*_E_ parameters can be used to forecast induced seismicity hazards under the assumption that these parameters are constant. We therefore investigate the impacts of temporal variations in *S*_I_ and *S*_E_ on the performance of these methods.

## 2. Case studies

In this study, we analyse the temporal evolution of *S*_I_ and *S*_E_ for cases of WWD-induced seismicity. We focus on WWD for several reasons:

— WWD has caused some of the most prominent cases of induced seismicity to date [25].
— WWD sequences often evolve over years-long or even decadal timescales, providing long time series over which temporal variations can be observed.
— The necessary injection datasets for WWD are often publicly available, in contrast to HF, where total well injection volumes may be available [27], but detailed injection time series are not.
— For HF, the location of injection changes with each frac stage along a horizontal well. Changes in *S*_I_ and *S*_E_ that are in fact generated by a spatial change in injection position could be misinterpreted as a temporal change within the same perturbed volume [13,20].
— The long-term, low rate, but ultimately high volume, nature of WWD provides a useful analogue to anticipated future activities, such as CCS, NGS and hydrogen storage, that are thought necessary to meet energy sustainability and energy security objectives [25,28–30].

**Figure 1.**
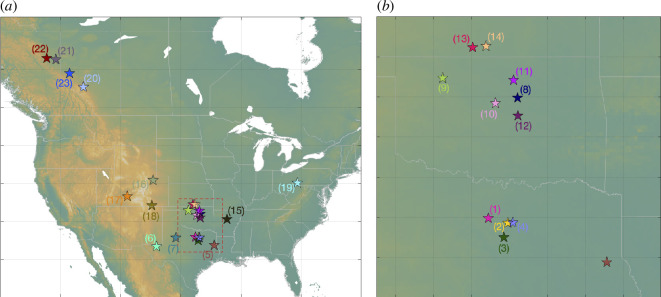
Map of case study locations across North America. (*b*) The area within the red dashed box in (*a*), with cases in northern Texas, Oklahoma and southern Kansas. Case numbers correspond to table 1, and the colours used to mark each case correspond to the colours in the subsequent figures.

Watkins *et al*. [25] compiled a database of WWD-induced seismicity case studies. Our cases, listed in table 1, are drawn from this database, with the additional criterion that injection rate time series must also be available for analysis. Sources for injection well data for each site are described in the electronic supplementary material. Figure 1 shows an overview map of our case study sites. Maps for each site, including earthquakes and injection wells, are provided in the electronic supplementary material, along with timelines showing the combined injection volumes and the seismicity.

**Table 1.** List of case sites used in our study.

	site	year of onset	*M*_MAX_	*M*_MIN_	no. of events	references
1	Azle-Reno	2013	3.6	0.8	634	Hennings *et al*. [31]
2	Dallas Fort Worth	2008	3.2	1.5	64	Hennings *et al*. [31]
3	Venus	2009	4.0	0.0	917	Hennings *et al*. [31]
4	Irving	2014	3.9	2.2	818	Hennings *et al*. [31]
5	Timpson	2008	4.8	2.1	49	Frohlich *et al*. [32]
6	Reeves	2018	4.9	1.3	208	Skoumal *et al*. [33]
7	Cogdell	2006	4.3	2.5	285	Gan and Frohlich [34]
8	Cushing	2013	5.0	2.5	501	McGarr and Barbour [35]
9	Fairview	2014	5.1	2.3	2711	Goebel *et al*. [36]
10	Guthrie	2011	4.2	2.5	1993	Schoenball *et al*. [37]
11	Pawnee	2013	5.8	2.2	1525	Walter *et al*. [38]
12	Prague	2009	5.7	2.2	1014	Keranen *et al*. [1]
13	Harper	2014	4.3	2.0	466	Verdecchia *et al*. [39]
14	Milan	2014	4.9	1.6	277	Verdecchia *et al*. [39]
15	Guy-Greenbrier	2009	4.7	2.1	1312	Horton [40]
16	Greeley	2014	3.3	0.5	1241	Yeck *et al*. [41]
17	Paradox	1991	4.4	1.5	6120	Block *et al*. [42]
18	Raton	1995	5.3	2.6	642	Nakai *et al*. [43]
19	Youngstown	2011	4.1	1.3	282	Kim *et al*. [44]
20	Cordel	1992	4.0	2.2	124	Schultz *et al*. [45]
21	Eagle West	1984	4.3	2.5	91	Horner *et al*. [46]
22	Graham	2003	4.0	2.3	246	Hosseini and Eaton [47]
23	Musreau	2018	3.9	1.7	44	Li *et al*. [48]

See electronic supplementary materials and Watkins *et al*. [25] for further details for each site.

In some cases, induced seismicity can be clearly linked to WWD into a single well, in which case the injection volume time series, Δ*V*(*t*), is easily established. In other areas, especially those with a high density of disposal wells, it can be challenging to determine which wells may be contributing to the seismicity, and therefore which should be included to create a compiled Δ*V*(*t*) time series. Based on observations of lateral distances for triggering of seismicity [28], for sequences with a large number of potentially associated wells, we adopt a relatively broad criterion of including any disposal well within 20 km of the induced seismicity sequence. We assess the sensitivity of our results to this distance in the electronic supplementary materials.

## 3. Method

For each case, we generate a time series for the number of events (larger than *M*_MIN_), the seismic moment released and the total injected volume. These time series form the basis of our subsequent analysis. We take *M*_MIN_ and G–R *b* values for each earthquake catalogue from Watkins *et al*. [25].

We perform measurements of *S*_I_ and *S*_E_ at 3-month intervals, starting at the first time window in which seismicity was recorded at a given site. Heretofore, measurements of *S*_I_ and *S*_E_ have typically been made on a cumulative basis: at a given time *t*, the value of *S*_I_ or *S*_E_ is computed from the total cumulative seismicity and the total cumulative injected volume at that time. Hereafter, we refer to values computed cumulatively as *S*_IT_ and *S*_ET_. Since in some cases, injection has taken place for many years prior to the onset of seismicity, for the cumulative volumes, we use volumes injected from a time 90 days prior to the first observed seismicity.

Measurements of *S*_IT_ and *S*_ET_ using cumulative time series may not perform well in capturing temporal changes in these parameters. Hence, we also perform time-windowed analysis, where the values of *S*_I_ and *S*_E_ at a given time *t* are computed using seismicity and injection volumes within a time window from (*t –* d*t*) to *t*. Hereafter, we refer to time-windowed values as *S*_IW_ and *S*_EW_. Determining an appropriate time window length, d*t*, in each case is challenging and dependent on the resolution of the dataset: too short a window will have low statistical power due to having a small number of events within any given window, while too long a window will smooth out the trends we hope to identify. The choice of d*t* used in our analysis is listed in the electronic supplementary materials and is varied depending on the duration of and the number of events within each earthquake catalogue.

One of our objectives in this study is to assess whether there are patterns of behaviour that are common across a wide range of injection cases. Different cases have experienced widely varying levels of induced seismicity, and as a result, produce values of *S*_I_ and *S*_E_ that vary across multiple orders of magnitude [22]. To make comparisons between such cases, we define normalized values, *S*_ITn_, *S*_IWn_, *S*_ETn_ and *S*_EWn_, where each time series is defined relative to the maximum value of that time series, such that:


(3.1) $$S_{\left[I,E\right]\left[T,W\right]n}=S_{\left[I,E\right]\left[T,W\right]}-\max\left(S_{\left[I,E\right]\left[T,W\right]}\right).$$

Note that this normalization does not perform any rescaling of the *S*_I_ and *S*_E_ time series, simply a shift in values such that each time series has a maximum value of 0. We also normalize the time axis along which these normalized values are computed, such that *t′* ranges from 0 to 1, representing the beginning and end of the time series.

## 4. Results

**Figure 2.**
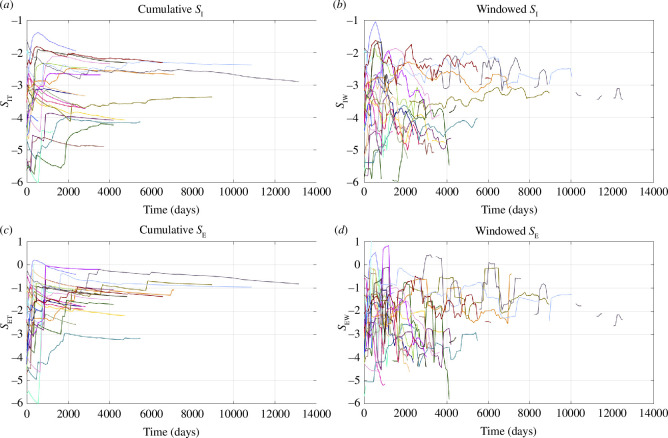
(*a*–*d*) Time evolution of the cumulative and windowed values of *S*_I_ and *S*_E_ for all our case studies. The colours of the lines correspond to the colours of the stars shown in figure 1.

**Figure 3.**
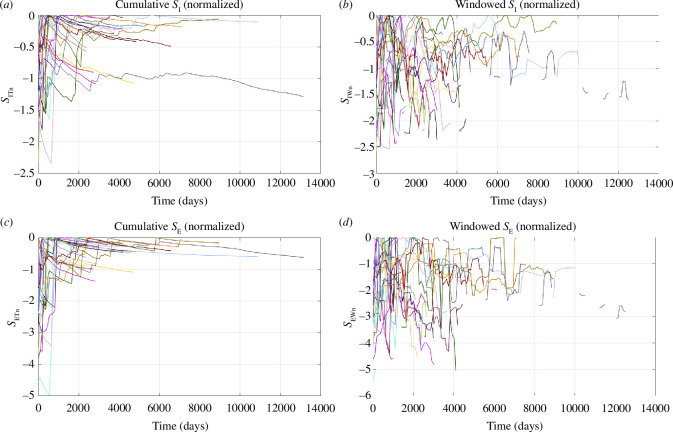
(*a*–*d*) Normalized values of *S*_I_ and *S*_E_ for all our case studies. The colours of the lines correspond to the colours of the stars shown in figure 1.

Figure 2 shows the time evolution of windowed and cumulative *S*_I_ and *S*_E_ values for each of our case study sites. Figure 3 shows the values of *S*_I_ and *S*_E_ when normalized to their respective maxima. Curves for *S*_I_ and *S*_E_ for each individual case are provided in the electronic supplementary materials.

### (a) Evolution of cumulative values

We begin by examining the behaviour of the cumulative time series (*S*_IT_ and *S*_ET_) as these can be more easily identified from visual inspection of figures 2 and 3. In all cases, the values of *S*_IT_ and *S*_ET_ rise steeply as each sequence of induced seismicity initiates. This acceleration usually occurs within 1000 days of the onset of the seismicity sequence (note that this is the time from the first observed seismicity at a site, not the start of injection, which in some cases may have been ongoing for many years before the onset of any observed seismicity). After this period, the cumulative *S*_IT_ and *S*_ET_ values stabilize and remain relatively constant throughout the remainder of each of the sequences. This behaviour is particularly apparent in figure 3*a,c*, which shows the cumulative values normalized to their respective maxima (*S*_ITn_ and *S*_ETn_). The *S*_ITn_ and *S*_ETn_ values rapidly reach their maxima, after which they continue forward at values of roughly *S*_ITn_ and *S*_ETn_ = 0.

**Figure 4.**
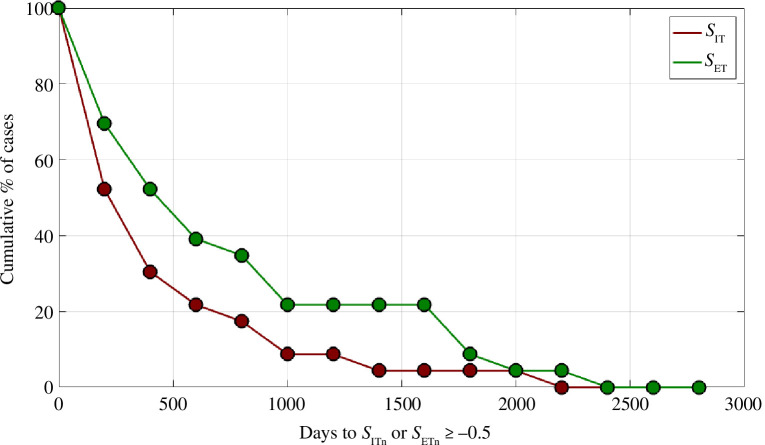
Cumulative histograms for the number of days from the start of each sequence until *S*_ITn_ (red) or *S*_ETn_ (green) reaches ≥ −0.5 (i.e. within 0.5 units of their respective maximum values). Values show the number of cases for which *t*(*S*_[I,E]T*n*_ ≥ −0.5) ≥ *t*. Frequencies are normalized to a percentage of cases.

We further investigate this behaviour in figure 4. We evaluate the time (in days) for *S*_ITn_ and *S*_ETn_ to reach a value ≥ −0.5. In other words, the number of days after the onset of seismicity at which *S*_IT_ and/or *S*_ET_ reach within 0.5 units of the maximum value it will ever reach during the entire sequence. Figure 4 shows a cumulative histogram (with frequencies normalized to a percentage) of the number of cases for which *t*(*S*_[I,E]T_ *_n_* ≥ −0.5) is greater than a given time.

We see that *S*_IT_ shows particularly rapid stabilization: for 70% of cases the cumulative *S*_I_ values reach within 0.5 units of the maximum they ever reach within 1 year of the onset of seismicity. For only two cases has the cumulative *S*_IT_ value not reached within 0.5 units of its ultimate maximum within 3 years of the onset of seismicity. The cumulative *S*_ET_ values take slightly longer to stabilize: 50% of cases have reached within −0.5 units of their respective maxima within 1 year, with 78% of cases reaching this value within 3 years.

### (b) Evolution of time-windowed values

**Figure 5.**
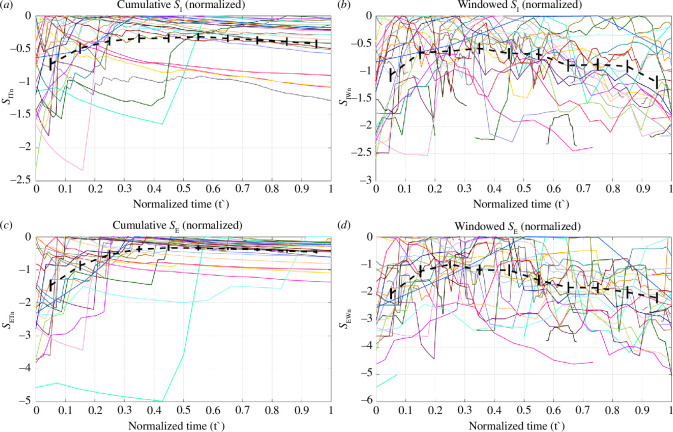
(*a*–*d*) Normalized values of *S*_I_ and *S*_E_ for all our case studies. The colours of the lines correspond to the colours of the stars shown in figure 1. The black lines show the average values as a function of time, with the error bars showing the standard error.

The time-windowed *S*_IW_ and *S*_EW_ values are inherently more variable and unstable, which is expected as each window contains a much smaller portion of seismicity and injection data when compared to the cumulative calculations. Hence, we see significant increases and decreases in *S*_IW_ and *S*_EW_ between time windows. This makes it harder to identify common trends and behaviours from a visual inspection of the time series. To address this, in figure 5, we normalize the time axis for each case, and then compute the average normalized *S*_IWn_ and *S*_EWn_ values as a function of normalized time (with the error bars in figure 5 representing the standard error, $s.e.=\sigma /\sqrt{n}$ ). These averages (dashed black line in figure 5*b,d*) allow us to identify common trends. We see that the averaged *S*_IWn_ and *S*_EWn_ values reach a maximum after the lapse of between 25% and 35% of the total sequence duration, after which the average values steadily decrease for the remainder of the sequence.

Watkins *et al*. [25] made a similar observation, finding that the largest earthquakes typically occurred within the first 20%–40% of the overall observed sequence (fig. 5 of [25]). However, since Watkins *et al*. did not examine injection rates, they were not able to establish whether this apparent peaking of the seismicity was in fact driven by changes in injection rates. The results presented here show that this behaviour is in fact driven by variations in the scaling with injection rates over time: we see that the scaling between injection rates and induced seismicity initially grows but then typically stabilizes within a few hundred days of the onset of seismicity, after which it begins to decay.

### (c) Time lags between injection and seismicity

The injection and seismicity time series that we have collated also allows us to examine any time lags between injection and the resulting seismicity. Several studies have identified systematic time delays between injection and the resulting seismicity, which is typically related to the times needed for pore pressure changes to propagate from the injection point to the critically stressed fault (or faults) that reactivate [49–51].

We assessed the time lags between injection and seismicity by computing the normalized correlation coefficients between the injection volumes and numbers of earthquakes (with magnitudes ≥ *M*_MIN_) within each time window, as a function of the lag between the time series. A positive time lag implies the seismicity lags the injection. Cross-correlation coefficients as a function of time lag are shown for every case in electronic supplementary material, figure S2. The time lag at which the cross-correlation coefficient is maximized, *λ*_maxXC_, is taken as indicating the time lag between injection and seismicity for each case.

**Figure 6.**
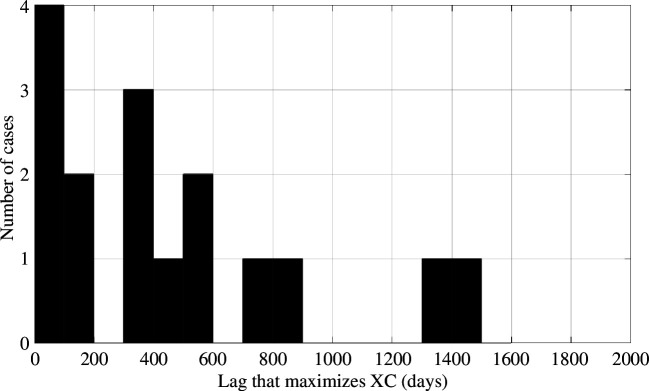
Histogram of the time lag values at which the normalized cross-correlation between injection volumes and rates of seismicity is maximized, *λ*_maxXC_. A positive time lag implies the seismicity is lagging the injection.

We found negative *λ*_maxXC_ values (i.e. where the injection appears to lag the seismicity) for seven cases. Clearly, these values have no physical basis since there is no mechanism by which the injection can lag the seismicity. Figure 6 shows a histogram for the remaining 16 cases with positive *λ*_maxXC_ values. The modal value is a time lag of less than 100 days, implying that rates of seismicity are closely following changes in injection. However, *λ*_maxXC_ values of between 300 and 600 days are also common. These results are consistent with the observations shown in figure 4, which show that the timescales in which the cumulative *S*_IT_ and *S*_ET_ values approach their peak are typically within 1–3 years of the onset of seismicity. This would be expected if these are the typical timescales required for the pressures at nearby faults to increase to the levels required to begin triggering seismicity. This distribution of time lags is also consistent with that simulated by Schultz *et al*. [52] to produce Båth’s law trailing seismicity.

## 5. Induced seismicity forecasting

Equations (1.5)–(1.8) describe how observations of *S*_I_ and *S*_E_ can be used to forecast the expected maximum magnitudes during induced seismicity sequences. In this section, we apply these methods in order to evaluate their respective performances. Previously, forecasting using *S*_I_ or *S*_E_ has been done using cumulative values as injection and seismicity progresses [13,20,53,54]. Here, we also use the time-windowed *S*_IW_ and *S*_EW_ values to perform forecasting.

We perform the forecasting using the same 3-month intervals over which we computed *S*_I_ and *S*_E_ values. To compute the modelled largest event magnitude, *M* ^M^_MAX_, for a given interval *t_i_*, we need to estimate the total number of events or the total seismic moment that will have been generated by the end of this interval. We do this by adding the modelled incremental number of events (or seismic moment) to the observed total number of events (or cumulative seismic moment) that has occurred prior to this time interval. For *S*_I_,


(5.1) $$N_{E\left(0\to t_{i}\right)}=N_{E\left(0\to t_{i-1}\right)}+\Delta V_{\left(t_{i}\right)}10^{S_{I\left[T,W\right]\left(t_{i-1}\right)}-bM},$$

where $N_{E(0\to t_{i})}$ is the modelled total number of events that will occur by the end of time interval *t_i_*, $N_{E(0\to t_{i-1})}$ is the total number of events that have been observed prior to time interval *t_i_*, ${∆V}_{\left(t_{i}\right)}$ is the planned injection volume for time interval *t_i_*, and $S_{I\left[T,W\right]\left(t_{i-1}\right)}$ is the cumulative or time-windowed *S*_I_ value measured during the previous time interval. The most likely largest magnitude event to have occurred up to the end of time interval *t_i_* is then given by [55]:


(5.2) $$M_{MAX}^{M}=M+\frac{1}{b}\log_{10}N_{E\left(0\to t_{i}\right)}.$$

As described in equation (1.2), we adopt the *M*_MIN_ value for each sequence as the reference magnitude *M*.

The equivalent steps for *S*_E_ are that we model the incremental seismic moment for time interval *t_i_* to estimate the total seismic moment that will be released by the end of this time interval:


(5.3) $$\Sigma M_{0\left(0\to t_{i}\right)}=\Sigma M_{0\left(0\to t_{i-1}\right)}+\mu \Delta V_{\left(t_{i}\right)}10^{S_{E\left[T,W\right]\left(t_{i-1}\right)}},$$

where ${\Sigma M}_{0(0\to t_{i-1})}$ is the total seismic moment release observed prior to time interval *t_i_* and $S_{E\left[T,W\right]\left(t_{i-1}\right)}$ is the cumulative or time-windowed *S*_E_ value measured during the previous time interval. The modelled total seismic moment release ${\Sigma M}_{0(0\to t_{i})}$ at the end of this time interval is then used as the input to equation (1.8) to compute *M* ^M^_MAX_.

**Figure 7.**
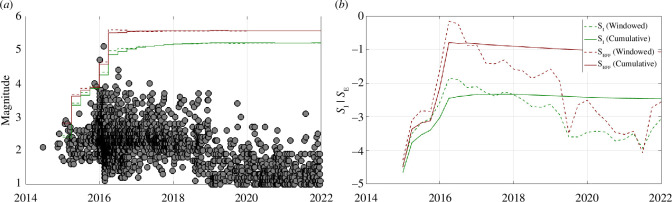
Example of our *M* ^M^_MAX_ forecasting approach. (*a*) shows the observed event magnitudes (grey circles) and the forecast magnitudes for each 3-month time window using the cumulative *S*_IT_ (solid green), time-windowed *S*_IW_ (dashed green), cumulative *S*_ET_ (solid red) and time-windowed *S*_EW_ (dashed red). Where a time window contains a new largest event, the largest event within that window is marked with a blue dot. (*b*) tracks the cumulative and time-windowed *S*_I_ and *S*_E_ values.

We assess the performance of our modelled *M* ^M^_MAX_ values by comparison with the observed magnitudes. Previous assessments of forecasting models have tended to focus on the largest overall event within the sequence [13,20]. However, it is of relevance to assess the performance of these methods as each sequence develops. Hence, whenever a given time window contains a new largest event (or events), then we compare the modelled *M* ^M^_MAX_ values for that time window with the largest observed event magnitude, *M* ^O^_MAX_, during that time window. An example of this process is depicted in figure 7 for the Fairview case study. Timelines of *M* ^O^_MAX_ forecasts relative to the observed seismicity are provided individually for each site in the electronic supplementary material.

**Figure 8.**
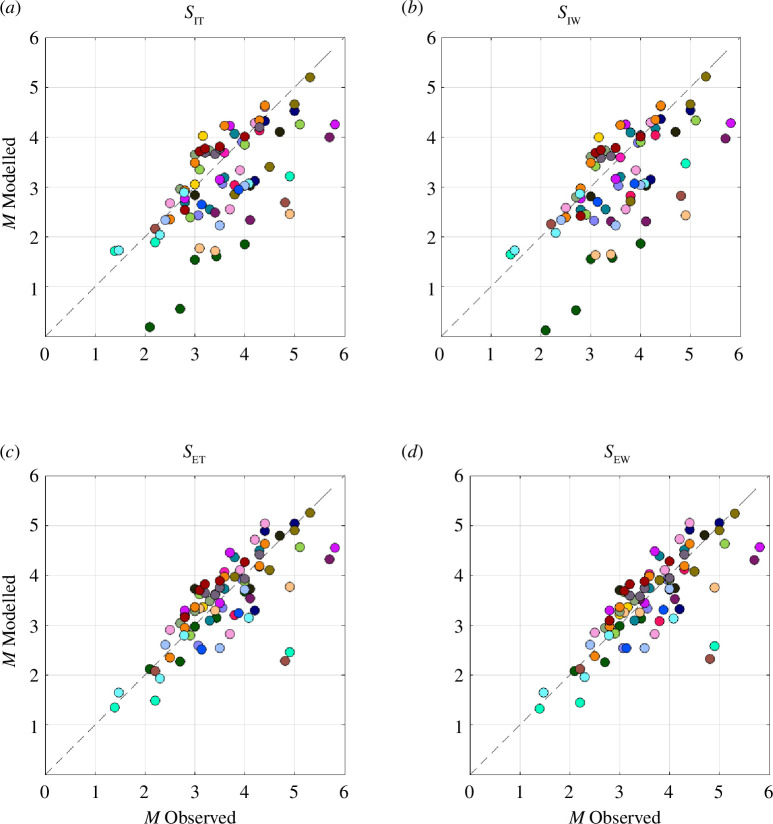
Comparison of observed and modelled maximum magnitudes during each sequence. The colours of the dots correspond to the colours for each case study used in figure 1. We show the results using (*a*) the cumulative *S*_IT_, (*b*) the time-windowed *S*_IW_, (*c*) the cumulative *S*_ET_ and (*d*) the time-windowed *S*_EW_. The dashed lines show a 1:1 relationship, which is the objective for the modelling.

We have a total of four forecast methods: using either *S*_I_ or *S*_E_, using either cumulative or time-windowed values in each case. The comparisons between *M* ^M^_MAX_ and *M* ^O^_MAX_ for all four methods are shown in figure 8. In all cases, we see a positive correlation between modelled and observed magnitudes, indicating that the models do provide useful predictive information. We quantify the models’ performance with RMS errors, *σ*_RMS_, and Pearson correlation coefficients, *ρ*, between *M* ^M^_MAX_ and *M* ^O^_MAX_ (table 2). We also compute the gradient of the line of (least squares) best fit, *m*, between observed and modelled magnitudes—for a well-performing model, this line should be close to 1. In many applications, we anticipate these models being used to guide decision-making during operations to avoid unwanted large events. Hence, we seek a model that does not produce underpredictions, where the actual magnitude significantly exceeds the preceding model values. Hence, we also compute *N*_UP_, the percentage of cases where the modelled value was a significant underprediction with *M* ^M^_MAX_ < *M* ^O^_MAX_ – 0.5.

**Table 2.** Performance metrics for the forecasting models based on the cumulative and time-windowed *S*_I_ and *S*_E_ values.

model	RMS	*ρ*	*m*	*N*_UP_ (*%*)
*S*_IT_	0.89	0.65	0.61	37.5
*S*_IW_	0.91	0.65	0.52	36.3
*S*_ET_	0.61	0.76	0.88	18.8
*S*_EW_	0.60	0.77	0.83	18.8

For both the *S*_I_ and *S*_E_ models, we find little difference in model performance between the cumulative and time-windowed models. However, there is a significant difference in performance between the *S*_I_ and *S*_E_ models, with the RMS errors and correlation coefficients indicating that the *S*_E_ approach provides a better match to the observed magnitudes. The *S*_E_ models also produced a line of best fit closer to 1, and fewer cases where the modelled values were significant underpredictions.

For both modelling approaches, where there are differences between modelled and observed magnitudes, the tendency is for the models to underpredict magnitudes. We note that for both *S*_I_ and *S*_E_ models, we have computed the most likely maximum event magnitude. This contrasts with previous studies where these methods have been used during active operations to manage induced seismicity [13,20]. In those papers, the upper 95% uncertainty limit was used, providing a larger margin between the forecast magnitudes and the observed seismicity. This was done to help the operators ensure that they did not reach unacceptable levels of seismicity. Using a higher uncertainty bound would systematically shift the *M* ^M^_MAX_ values in figures 7 and 8 upwards. This could reduce the RMS errors and would reduce the number of underpredictions, but would not change the scatter (as measured by the correlation coefficient) or the gradient of the best-fit line between observed and modelled magnitudes.

The underpredicted magnitudes tend to be found where a rapid acceleration in seismicity takes place. Figure 7 shows an example of this. In early 2016, the levels of seismicity in the Fairview sequence accelerated sharply. This is reflected in *S*_I_ and *S*_E_ values, which also increase rapidly at this time. However, for a given time window, the *M* ^M^_MAX_ forecasts are based on *S*_I_ and *S*_E_ values from the previous time step. Given the sharp acceleration in seismicity, the earlier values are substantially lower (by orders of magnitude), which then leads to an underpredicted *M* ^M^_MAX_ forecast.

Kettlety *et al*. [20] found a similar issue when using *S*_E_ to forecast induced seismicity during HF. As the volume of rock affected by the HF grew, more faults began to be reactivated. Some of the later faults to be reactivated proved to be more seismogenic than the first faults to be reactivated. As a result, the *M* ^M^_MAX_ forecasts based on *S*_E_ measurements made during earlier phases of the HF underpredicted the levels of seismicity as the new, more seismogenic faults began to activate.

We hypothesize that this issue may apply to many of our sequences as well. Various factors may influence the seismogenic potential of faults, e.g. their orientation within the *in situ* stress field [20,56] or their frictional properties [57]. As the pore pressure perturbation spreads from the injection point (or points), it may encounter and reactivate faults further from the well. If these faults are more seismogenic then the levels of seismicity will increase, and therefore forecasts based on *S*_I_ or *S*_E_ values measured earlier in the sequence will produce underpredictions.

Verdon and Bommer [26] and Watkins *et al*. [25] applied the Next Record Breaking Event (NRBE) forecasting method [58] to sequences of HF and WWD-induced seismicity. They concluded that the NRBE approach had clear utility as a forecasting method to guide operational decision-making. However, in some instances the observed seismicity significantly exceeded the forecast values, meaning that the method cannot be used as an absolute guarantee that larger events will not occur. We reach similar conclusions here for the volume-based forecasting methods. For example, at the Reeves sequence, the *S*_E_ forecast values were at *M* 2.5 when the *M* 4.9 event occurred, and at Timpson, the *S*_E_ forecast values were at *M* 2.3 when the *M* 4.8 event occurred. Hence, while these forecasting methods have clear utility, as demonstrated by the statistically significant correlation between observed and modelled magnitudes, the occurrence of events that are significantly larger than the forecast values cannot be precluded entirely.

## 6. Discussion

### (a) Scaling between injection rate and pore pressures

In §1.3, we described the geomechanical assumptions that underpin the expectation that rates of induced seismicity will scale linearly with the injection rate. A key assumption is that the injection rate provides a reasonable proxy for the stressing rate in the subsurface since equation (1.1) defines a linear scaling between the rate of seismicity and the stressing rate. For injection-induced seismicity, the primary driver for triggering earthquakes is typically the associated increase in pore pressure, which causes a reduction in effective normal stresses. Hence, the relevant stressing rate is the change in pore pressure, Δ*P*. The scaling between the injection rate, Δ*V*, and the resulting change in pore pressure, Δ*P*, will depend on the specific conditions within the reservoir.

We investigate this scaling further using some simple, generic reservoir simulations. These simulations are not intended to represent any single case study or scenario, but they provide a reasonable approximation for typical conditions in which deep WWD takes place. We use the commercial reservoir simulation code Tempest [59] to simulate the injection of water into a deep reservoir. Table 3 lists the key reservoir parameters in our simulations. Each simulation consists of water injection via a single well in the centre of a cuboid reservoir with a thickness of 100 m and lateral dimensions of *R_x_* × *R_x_*, where we vary *R_x_* from 10 to 30 km (Models 1–5), with an additional model where the volumes of the cells at the edges of the reservoir are infinite, essentially creating a reservoir that is unbounded.

**Table 3.** Parameter values for our reservoir simulations.

parameter	value	model no.	lateral dimensions (*R_x_*)
injected fluid	water	1	10 × 10 km
initial reservoir fluid	water	2	12 × 12 km
reservoir depth	2500 m	3	15 × 15 km
reservoir thickness	100 m	4	20 × 20 km
initial pressure	hydrostatic	5	30 × 30 km
porosity	0.2	6	unbounded
vertical permeability	0.1 D		
lateral permeability	1 D		
rock bulk modulus	16 GPa		
grid cell size	50 × 50 × 10 m		
injection rate	1000 m^3^/day		

Our motivation for doing so is that the modelled pressure change produced by injection is strongly dependent on the boundary conditions and in particular, the bounding dimensions of the reservoir. In some cases, reservoirs may be bounded by faults that create hydraulic barriers to flow, or by stratigraphic changes in reservoir properties (e.g. a high permeability stratum being pinched out by surrounding low permeability formations). Many of the formations targeted for WWD in North America are very extensive laterally [60]. However, in such situations, the ‘bounds’ of the reservoir could be taken as representative of the distances between injection wells (or more specifically, the mid-point therebetween). In each model, water is injected via the single well at a fixed rate of 1000 m^3^/day for a period of 3000 days.

**Figure 9.**
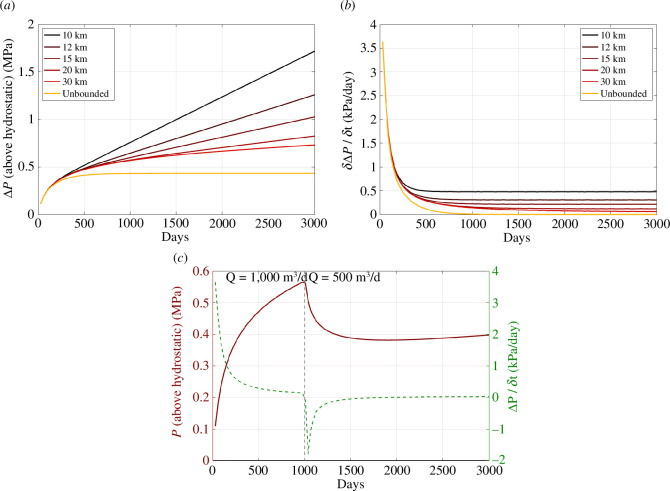
Reservoir pore pressures (*a*) and the rate of pressure change (*b*) at a distance of 1 km from the injection well for our modelled reservoir scenarios. In (*c*), we plot pore pressures (red) and rate of pressure change (green dashed line) for a model where the injection rate is reduced from 1000 to 500 m^3^/day at *t* = 1000 days.

The resulting modelled pressures at a distance of 1 km from the well are plotted in figure 9. This position is chosen arbitrarily to demonstrate the response of pore pressures within the reservoir at a reasonable distance from the near-well environment. Evidently, pressure changes will be larger, and occur sooner, at shorter distances from the well, and vice versa for longer distances. Figure 9*a* shows pressure increases relative to the initial hydrostatic conditions. Figure 9*b* shows the rates of pressure change, *δ*Δ*P*/*δt*. For roughly the first year of injection, the pressures follow a similar trajectory irrespective of the bounding conditions. The rates of pressure increase are the largest at this time. For the bounded reservoir cases, the pressure increase is linear thereafter, with the rate of increase controlled by the dimensions of the reservoir bounds, where the rate of increase is higher for smaller reservoirs. After approximately 2 years, the ‘unbounded’ case reaches a steady-state condition with no further pressure increase, as the flow out of the reservoir edges matches the rate at which fluid is injected.

Our model results provide a useful context within which our observations of seismicity rate changes can be examined. Our simulations are representative of generic, typical WWD conditions, they are not intended to be an explicit representation of any particular site—the generation of detailed reservoir simulations for each case study site is beyond the scope of this article. Nevertheless, it is reasonable to expect that our various cases will sit qualitatively somewhere within the range bounded by our model suite. From equation (1.1), we expect the rate of induced seismicity to scale with the rate of pore pressure change, δΔ*P*/δ*t*, as plotted in figure 9*b*. These models suggest we should expect an initial acceleration of seismicity as pore pressures increase more sharply during the early phases of injection, followed by reducing levels of seismicity as δΔ*P*/δ*t* decreases and stabilizes. This behaviour is matched in a qualitative sense by our observed seismicity sequences, where most cases show an initial acceleration in induced seismicity, followed by a reduction and stabilization. This match suggests that rates of pressure change are indeed the driving factor in controlling the rates of induced seismicity. This being the case, it may be possible to produce more accurate forecasts of induced seismicity hazard if we directly calibrate rates of seismicity to rates of pressure change, rather than using injection rates as a proxy for the pressure change [21,61].

### (b) Influence of actions taken to mitigate induced seismicity

In some of the cases we have studied, actions to mitigate the levels of induced seismicity have been taken by operators of these sites (or have been mandated by regulators). For example, since the mid-2010s, the Oklahoma Corporation Commission has mandated reductions of up to 40% in the volumes of wastewater being disposed [62]. For the Paradox Valley case, the injection programme has included regular pauses in injection to allow pore pressures to dissipate [63]. At Greeley, after the onset of seismicity, the operator cemented the lower part of the injection well to divert pore pressure increases away from the more seismogenic basement strata [41]. Clearly, these actions may be responsible for some of the reduction and stabilization of induced seismicity rates that we have observed.

We note that the behaviour we have described appears to be fairly ubiquitous irrespective of whether or not mitigating actions have been taken. That is not to say that mitigating actions are unnecessary, as such actions will have caused the levels of seismicity to drop sooner and by a larger degree than might otherwise have been the case. However, the changes in seismicity rates we observe are, via the *S*_I_ and *S*_E_ parameters, normalized to the injection rates. Hence, in cases where injection volumes have been reduced in response to seismicity, the decreases in seismicity do not simply represent a decrease in injection rate, with the seismicity continuing to scale at the same rate with respect to injection. Instead, the decreases in *S*_I_ and *S*_E_ we observe represent decreases in seismicity rates that are proportionally larger than the decrease in injection rate.

Incidentally, we note that if it is the case that the mitigating actions have been successful in stopping or reducing the seismicity rates, then this is clearly encouraging with respect to our overall ability to manage and mitigate induced seismicity during large-scale injection projects. Experiences with mitigating induced seismicity at WWD sites will therefore be of direct relevance for future large-scale injection industries such as CCS.

The fact that induced seismicity rates might be more properly scaled with rates of pressure change, rather than rates of injection, is a salient issue here since the impact of many of the mitigation actions will be to produce a reduction in reservoir pore pressures relative to injection rates. To investigate this, we produce an additional reservoir injection model in which a reduction in injection rates takes place mid-way through the injection period. In this case, we use the 30 km bounded model (Model 5) and reduce the injection rate from 1000 to 500 m^3^/day after a period of 1000 days. The resulting pressure changes are shown in figure 9*c*. We see that the absolute pressures drop in response to the drop in injection rate, and never again approach the levels seen during the higher-rate injection. The rates of pore pressure change, *δ*Δ*P*/*δt*, become negative, they do not become positive again until almost 1000 days after the reduction in injection rate, and they remain significantly smaller than those for the constant injection rate cases.

We stress again that these are generic models, which are not intended to represent any specific site or actual mitigation action. Nevertheless, the modelled changes in pressure relative to the change in injection rate—where a 50% reduction in rates actually leads to the rate of pressure change becoming negative—shows why we might not expect rates of pore pressure change, and therefore according to equation (1.1), the rates of seismicity, to directly scale with injection rates. This further demonstrates how more accurate forecasts of induced seismicity hazard may require models where seismicity rates are scaled to rates of pressure change, rather than injection rates. Moreover, such models could be used, for sites that are experiencing unacceptable levels of induced seismicity, to investigate the extent to which different mitigating actions would reduce the levels of ongoing induced seismicity.

We note that this approach to modelling induced seismicity generation implies that seismicity will stop immediately when pore pressures drop. In contrast, we know that trailing seismicity often occurs after the cessation of injection [26]. Few cases of trailing seismicity have been observed for WWD into large, extensive aquifers, although this could be considered a semantic issue since there are few examples disposal of operations of this kind where the injection has been stopped suddenly [25]. No events can be called trailing events if the injection is never stopped.

Observations of trailing seismicity show that they often follow similar behaviours to tectonic aftershocks, following Båth’s law [52] and showing Omori-Utsu temporal decay [64]. This suggests that trailing seismicity is primarily driven by similar processes to tectonic aftershocks, such as static and dynamic stress transfer between events and transfer of pore pressures between asperities on fault planes, for example. Hence, a more comprehensive model might incorporate an underlying rate of seismicity that is scaled to the rate of pressure change, with additional terms that describe the trailing events in a manner that is similar to aftershock nucleation in tectonic settings.

## 7. Conclusions

We have compiled time series of fluid injection and induced seismicity rates for over 20 cases of WWD-induced seismicity in North America. We use these time series to investigate the temporal evolution of the scaling between injection rates and seismicity, as quantified by the *S*_I_ and *S*_E_ parameters. We computed these parameters on both a cumulative and time-windowed basis. We find that the cumulative values typically show an initial increase before reaching a maximum value—this stabilization typically occurs within 1–3 years of the onset of seismicity. The time-windowed values showed more variability, which is to be expected given that they are computed from short time series. However, the time-windowed averages showed a clear pattern of behaviour, with values increasing during the early phases of injection, before stabilizing and reducing during the latter phases.

We use the observed scaling between injection volumes and seismicity rates to assess the performance of magnitude forecasting models. We find that models using either *S*_I_ or *S*_E_ both produce statistically significant correlation between observed and modelled event magnitudes, indicating that these methods do have predictive utility. We found little difference in performance between time-windowed and cumulative analyses. The *S*_E_ models produced slightly higher correlations and lower RMS errors than the *S*_I_ models.

We interpret the observed variations in seismicity rates with respect to the pressure changes produced by long-term injection into large, high permeability, relatively unbounded aquifers. During the initial stages of injection, the pore pressure perturbation will extend outwards from the well, reaching and reactivating more seismogenic faults and increasing the rates of seismicity. With time, in relatively unbounded aquifers, the rate of pore pressure increase will drop, leading to a reduction in the triggering of seismicity. Likewise, mitigating actions that reduce the rates of pressure increase may further reduce the rates of seismicity. We conclude that, where possible, changes in seismicity rates could be calibrated against site-specific models of pore pressure change. Such models could lead to more accurate forecasting of induced seismicity hazard, as well as allow the ability to simulate the extent to which different interventions might reduce the induced seismicity hazard.

## Data Availability

Earthquake catalogues (and associated parameters such as b values and magnitudes of completeness) were drawn from the electronic supplementary materials of Watkins *et al*. [25]. In turn, these earthquake catalogues were sourced from a variety of public earthquake databases and from the academic literature [25]. Injection datasets were drawn from a range of publicly available databases. Our sources for each site are described in the electronic supplementary materials. In addition, we provide (in a zip file) Matlab data files containing the data for each case, and two Matlab scripts to compute S_I_ and S_EFF_ values, and the resulting M_max_ values. The contents of the zip file are described in the electronic supplementary materials [65].

## References

[1] Keranen KM , Savage HM , Abers GA , Cochran ES . 2013 Potentially induced earthquakes in Oklahoma, USA: Links between wastewater injection and the 2011 Mw 5.7 earthquake sequence. Geology **41** , 699–702. (10.1130/G34045.1)

[2] Yeck WL , Hayes GP , McNamara DE , Rubinstein JL , Barnhart WD , Earle PS , Benz HM . 2017 Oklahoma experiences largest earthquake during ongoing regional wastewater injection hazard mitigation efforts. Geophys. Res. Lett. **44** , 711–717. (10.1002/2016GL071685)

[3] Ellsworth WL , Giardini D , Townend J , Ge S , Shimamoto T . 2019 Triggering of the Pohang, Korea, earthquake (Mw 5.5) by enhanced geothermal system stimulation. Seismol. Res. Lett. **90** , 1844–1858. (10.1785/0220190102)

[4] Lei X , Wang Z , Su J . 2019 The December 2018 ML 5.7 and January 2019 ML 5.3 earthquakes in South Sichuan Basin induced by shale gas hydraulic fracturing. Seismol. Res. Lett. **90** , 1099–1110. (10.1785/0220190029)

[5] Evensen D *et al* . 2022 Effect of linguistic framing and information provision on attitudes towards induced seismicity and seismicity regulation. Sci. Rep. **12** , 11239. (10.1038/s41598-022-15448-4) 35788650 PMC9253309

[6] Dieterich J . 1994 A constitutive law for rate of earthquake production and its application to earthquake clustering. J. Geophys. Res. **99** , 2601–2618. (10.1029/93JB02581)

[7] Shapiro SA , Dinske C , Langenbruch C , Wenzel F . 2010 Seismogenic index and magnitude probability of earthquakes induced during reservoir fluid stimulations. Lead. Edge **29** , 304–309. (10.1190/1.3353727)

[8] Hallo M , Oprsal I , Eisner L , Ali MY . 2014 Prediction of magnitude of the largest potentially induced seismic event. J. Seismol. **18** , 421–431. (10.1007/s10950-014-9417-4)

[9] Gutenberg B , Richter CF . 1944 Frequency of earthquakes in California. Bull. Seismol. Soc. Am. **34** , 185–188. (10.1785/BSSA0340040185)

[10] De Barros L , Cappa F , Guglielmi Y , Duboeuf L , Grasso JR . 2019 Energy of injection-induced seismicity predicted from in-situ experiments. Sci. Rep. **9** , 4999. (10.1038/s41598-019-41306-x) 30899030 PMC6428893

[11] Galis M , Ampuero JP , Mai PM , Cappa F . 2017 Induced seismicity provides insight into why earthquake ruptures stop. Sci. Adv. **3** , eaap7528. (10.1126/sciadv.aap7528) 29291250 PMC5744472

[12] McGarr A . 2014 Maximum magnitude earthquakes induced by fluid injection. J. Geophys. Res. Solid Earth **119** , 1008–1019. (10.1002/2013JB010597)

[13] Clarke H , Verdon JP , Kettlety T , Baird AF , Kendall JM . 2019 Real‐time imaging, forecasting, and management of human‐induced seismicity at Preston New Road, Lancashire, England. Seismol. Res. Lett. (10.1785/0220190110)

[14] Cesca S , Dost B , Oth A . 2013 Preface to the special issue “Triggered and induced seismicity: probabilities and discrimination.” J. Seismol. **17** , 1–4. (10.1007/s10950-012-9338-z)

[15] Park Y , Beroza GC , Ellsworth WL . 2022 Basement fault activation before larger earthquakes in Oklahoma and Kansas. Seismic Record **2** , 197–206. (10.1785/0320220020)

[16] McNamara DE , Benz HM , Herrmann RB , Bergman EA , Earle P , Holland A , Baldwin R , Gassner A . 2015 Earthquake hypocenters and focal mechanisms in central Oklahoma reveal a complex system of reactivated subsurface strike‐slip faulting. Geophys. Res. Lett. **42** , 2742–2749. (10.1002/2014GL062730)

[17] Eaton DW , Igonin N . 2018 What controls the maximum magnitude of injection-induced earthquakes? Lead. Edge **37** , 135–140. (10.1190/tle37020135.1)

[18] Kwiatek G *et al* . 2019 Controlling fluid-induced seismicity during a 6.1-km-deep geothermal stimulation in Finland. Sci. Adv. **5** , eaav7224. (10.1126/sciadv.aav7224) 31049397 PMC6494490

[19] Verdon JP . 2016 Using microseismic data recorded at the Weyburn CCS-EOR site to assess the likelihood of induced seismic activity. Int. J. Greenh. Gas Control **54** , 421–428. (10.1016/j.ijggc.2016.03.018)

[20] Kettlety T , Verdon JP , Butcher A , Hampson M , Craddock L . 2021 High-resolution Imaging of the ML 2.9 August 2019 earthquake in Lancashire, United Kingdom, induced by hydraulic fracturing during Preston New Road PNR-2 operations. Seismol. Res. Lett. **92** , 151–169. (10.1785/0220200187)

[21] Langenbruch C , Zoback MD . 2016 How will induced seismicity in Oklahoma respond to decreased saltwater injection rates? Sci. Adv. **2** , e1601542. (10.1126/sciadv.1601542) 28138533 PMC5262442

[22] Dinske C , Shapiro SA . 2013 Seismotectonic state of reservoirs inferred from magnitude distributions of fluid-induced seismicity. J. Seismol. **17** , 13–25. (10.1007/s10950-012-9292-9)

[23] Kao H , Hyndman R , Jiang Y , Visser R , Smith B , Babaie Mahani A , Leonard L , Ghofrani H , He J . 2018 Induced seismicity in Western Canada linked to tectonic strain rate: implications for regional seismic hazard. Geophys. Res. Lett. **45** , 11104–11115. (10.1029/2018GL079288)

[24] Rodríguez-Pradilla G , Eaton DW , Verdon JP . 2022 Basin-scale multi-decadal analysis of hydraulic fracturing and seismicity in western Canada shows non-recurrence of induced runaway fault rupture. Sci. Rep. **12** , 14463. (10.1038/s41598-022-18505-0) 36002601 PMC9402563

[25] Watkins TJM , Verdon JP , Rodríguez-Pradilla G . 2023 The temporal evolution of induced seismicity sequences generated by low-pressure, long-term fluid injection. J. Seismol. **27** , 243–259. (10.1007/s10950-023-10141-z)

[26] Verdon JP , Bommer JJ . 2021 Green, yellow, red, or out of the blue? An assessment of Traffic Light Schemes to mitigate the impact of hydraulic fracturing-induced seismicity. J. Seismol. **25** , 301–326. (10.1007/s10950-020-09966-9)

[27] Verdon JP , Rodríguez-Pradilla G . 2023 Assessing the variability in hydraulic fracturing-induced seismicity occurrence between North American shale plays. Tectonophysics **859** , 229898. (10.1016/j.tecto.2023.229898)

[28] Verdon JP . 2014 Significance for secure CO_2_ storage of earthquakes induced by fluid injection . Environ. Res. Lett. **9** , 064022. (10.1088/1748-9326/9/6/064022)

[29] Verdon JP , Kendall JM , Stork AL , Chadwick RA , White DJ , Bissell RC . 2013 Comparison of geomechanical deformation induced by megatonne-scale CO_2_ storage at Sleipner, Weyburn, and In Salah. Proc. Natl Acad. Sci. USA **110** , E2762–71. (10.1073/pnas.1302156110) 23836635 PMC3725089

[30] Zoback MD , Gorelick SM . 2012 Earthquake triggering and large-scale geologic storage of carbon dioxide. Proc. Natl Acad. Sci. USA **109** , 10164–10168. (10.1073/pnas.1202473109) 22711814 PMC3387039

[31] Hennings PH , Nicot J ‐P. , Gao RS , DeShon HR , Lund Snee J ‐E. , Morris AP , Brudzinski MR , Horne EA , Breton C . 2021 Pore pressure threshold and fault slip potential for induced earthquakes in the Dallas‐Fort Worth Area of North Central Texas. Geophys. Res. Lett. **48** , e2021GL093564. (10.1029/2021GL093564)

[32] Frohlich C , Ellsworth W , Brown WA , Brunt M , Luetgert J , MacDonald T , Walter S . 2014 The 17 May 2012 M 4.8 earthquake near Timpson, East Texas: an event possibly triggered by fluid injection . J. Geophys. Res. **119** , 581–593. (10.1002/2013JB010755)

[33] Skoumal RJ , Kaven JO , Barbour AJ , Wicks C , Brudzinski MR , Cochran ES , Rubinstein JL . 2021 The induced Mw 5.0 March 2020 West Texas seismic sequence. J. Geophys. Res. **126** , e2020JB020693. (10.1029/2020JB020693)

[34] Gan W , Frohlich C . 2013 Gas injection may have triggered earthquakes in the Cogdell oil field, Texas. Proc. Natl Acad. Sci. USA **110** , 18786–18791. (10.1073/pnas.1311316110) 24191019 PMC3839743

[35] McGarr A , Barbour AJ . 2017 Wastewater Disposal and the earthquake sequences during 2016 Near Fairview, Pawnee, and Cushing, Oklahoma. Geophys. Res. Lett. **44** , 9330–9336. (10.1002/2017GL075258)

[36] Goebel THW , Weingarten M , Chen X , Haffener J , Brodsky EE . 2017 The 2016 Mw5.1 Fairview, Oklahoma earthquakes: evidence for long-range poroelastic triggering at >40 km from fluid disposal wells. Earth Planet. Sci. Lett. **472** , 50–61. (10.1016/j.epsl.2017.05.011)

[37] Schoenball M , Walsh FR , Weingarten M , Ellsworth WL . 2018 How faults wake up: the Guthrie-Langston, Oklahoma earthquakes. Lead. Edge **37** , 100–106. (10.1190/tle37020100.1)

[38] Walter JI , Chang JC , Dotray PJ . 2017 Foreshock seismicity suggests gradual differential stress increase in the months prior to the 3 september 2016 M _w_ 5.8 pawnee earthquake. Seismol. Res. Lett. **88** , 1032–1039. (10.1785/0220170007)

[39] Verdecchia A , Cochran ES , Harrington RM . 2021 Fluid‐Earthquake and Earthquake‐Earthquake Interactions in Southern Kansas, USA. J. Geophys. Res. **126** , e2020JB020384. (10.1029/2020JB020384)

[40] Horton S . 2012 Disposal of hydrofracking waste fluid by injection into subsurface aquifers triggers earthquake swarm in Central Arkansas with potential for damaging earthquake. Seismol. Res. Lett. **83** , 250–260. (10.1785/gssrl.83.2.250)

[41] Yeck WL , Sheehan AF , Benz HM , Weingarten M , Nakai J . 2016 Rapid response, monitoring, and mitigation of induced seismicity near Greeley, Colorado. Seismol. Res. Lett. **87** , 837–847. (10.1785/0220150275)

[42] Block LV , Wood CK , Yeck WL , King VM . 2014 The 24 january 2013 ML 4.4 earthquake near Paradox, Colorado, and its relation to deep well injection. Seismol. Res. Lett. **85** , 609–624. (10.1785/0220130188)

[43] Nakai JS , Weingarten M , Sheehan AF , Bilek SL , Ge S . 2017 A possible causative mechanism of Raton Basin, New Mexico and Colorado earthquakes using recent seismicity patterns and pore pressure modeling. J. Geophys. Res. **122** , 8051–8065. (10.1002/2017JB014415)

[44] Kim WY . 2013 Induced seismicity associated with fluid injection into a deep well in Youngstown, Ohio. J. Geophys. Res. **118** , 3506–3518. (10.1002/jgrb.50247)

[45] Schultz R , Stern V , Gu YJ . 2014 An investigation of seismicity clustered near the Cordel Field, west central Alberta, and its relation to a nearby disposal well. J. Geophys. Res. **119** , 3410–3423. (10.1002/2013JB010836)

[46] Horner RB , Barclay JE , MacRae JM . 1994 Earthquakes and hydrocarbon production in the Fort St. John area of northeastern British Columbia. Can. J. Explor. Geophy. **30** , 39–50.

[47] Hosseini BK , Eaton DW . 2018 Fluid flow and thermal modeling for tracking induced seismicity near the Graham disposal well, British Columbia, Canada. In SEG technical program expanded abstracts 2018, pp. 4987–4991. (10.1190/segam2018-2996360.1)

[48] Li T , Gu YJ , Wang J , Wang R , Yusifbayov J , Canales MR , Shipman T . 2022 Earthquakes induced by wastewater disposal near Musreau Lake, Alberta, 2018–2020. Seismol. Res. Lett. **93** , 727–738. (10.1785/0220210139)

[49] Grigoratos I , Rathje E , Bazzurro P , Savvaidis A . 2020 Earthquakes induced by wastewater injection, part I: model development and hindcasting. Bull. Seismol. Soc. Am. **110** , 2466–2482. (10.1785/0120200078)

[50] Hsieh PA , Bredehoeft JD . 1981 A reservoir analysis of the Denver earthquakes: a case of induced seismicity. J. Geophys. Res. **86** , 903–920. (10.1029/JB086iB02p00903)

[51] Norbeck JH , Rubinstein JL . 2018 Hydromechanical earthquake nucleation model forecasts onset, peak, and falling rates of induced seismicity in Oklahoma and Kansas. Geophys. Res. Lett. **45** , 2963–2975. (10.1002/2017GL076562)

[52] Schultz R , Ellsworth WL , Beroza GC . 2022 Statistical bounds on how induced seismicity stops. Sci. Rep. **12** , 1184. (10.1038/s41598-022-05216-9) 35075145 PMC8786864

[53] Hajati T , Langenbruch C , Shapiro SA . 2015 A statistical model for seismic hazard assessment of hydraulic‐fracturing‐induced seismicity. Geophys. Res. Lett. **42** , 10601–10606. (10.1002/2015GL066652)

[54] Verdon JP , Budge J . 2018 Examining the capability of statistical models to mitigate induced seismicity during hydraulic fracturing of shale gas reservoirs. Bull. Seismol. Soc. Am. **108** , 690–701. (10.1785/0120170207)

[55] van der Elst NJ , Page MT , Weiser DA , Goebel THW , Hosseini SM . 2016 Induced earthquake magnitudes are as large as (statistically) expected. J. Geophys. Res. **121** , 4575–4590. (10.1002/2016JB012818)

[56] Walsh FR , Zoback MD . 2016 Probabilistic assessment of potential fault slip related to injection-induced earthquakes: application to north-central Oklahoma, USA. Geology **44** , 991–994. (10.1130/G38275.1)

[57] Allen M , Kettlety T , Faulkner D , Kendall JM , Fisher Q , Verdon JP . 2021 The relationship between earthquake size distributions and laboratory measured frictional stability parameters from induced seismicity on faults during fluid injection: AGU Fall Meeting Abstracts S22A–3.

[58] Cao NT , Eisner L , Jechumtálová Z . 2020 Next record breaking magnitude for injection induced seismicity. First Break **38** , 53–57. (10.3997/1365-2397.fb2020010)

[59] Emerson . 2014 Tempest reservoir engineering. See http://www.emerson.com/documents/automation/tempest-more-data-sheet-2014-en-82050.pdf (accessed 29 June 2023).

[60] Johnson KS . 1991 Geologic overview and economic importance of late Cambrian and Ordovician age rocks in Oklahoma. In Late Cambrian-Ordovician geology of the Southern Midcontinent, 1989 symposium: Oklahoma Geological Survey circular (ed. KS Johnson ), pp. 3–14, vol. **92** .

[61] Molina I , Velásquez JS , Rubinstein JL , Garcia-Aristizabal A , Dionicio V . 2020 Seismicity induced by massive wastewater injection near Puerto Gaitán, Colombia. Geophys. J. Int. **223** , 777–791. (10.1093/gji/ggaa326)

[62] OCC . 2016 Media advisory - regional earthquake response plan for western Oklahoma: Oklahoma Corporation Commission, Oklahoma City, OK. See https://oklahoma.gov/content/dam/ok/en/occ/documents/ajls/news/2016/02-16-16westernregionalplan.pdf (accessed 29 June 2023).

[63] Ake J , Mahrer K , O’Connell D , Block L . 2005 Deep-injection and closely monitored induced seismicity at Paradox Valley, Colorado. Bull. Seismol. Soc. Am. **95** , 664–683. (10.1785/0120040072)

[64] Mancini S , Werner MJ , Segou M , Baptie B . 2021 Probabilistic forecasting of hydraulic fracturing-induced seismicity using an injection-rate driven ETAS model. Seismol. Res. Lett. **92** , 3471–3481. (10.1785/0220200454)

[65] Verdon J , Pullen B , Rodríguez-Pradilla G . 2024 Supplementary material from: Growth and stabilisation of induced seismicity rates during long-term, low pressure fluid injection. Figshare. (10.6084/m9.figshare.c.7249278) PMC11363676 38910395

